# Effects of music therapy on COVID-19 patients’ anxiety, depression, and life quality: A protocol for systematic review and meta-analysis

**DOI:** 10.1097/MD.0000000000026419

**Published:** 2021-07-02

**Authors:** Xiaomei Chen, Haiying Li, Xiaoying Zheng, Jiaqi Huang

**Affiliations:** a Operating Room, Second Affiliated Hospital of Hainan Medical College, Haikou 570311; b Operating Room, Hainan Provincial Children's Hospital, Haikou 570100; c Cross Infection Control Office, Hospital Office of Hainan Provincial Anning Hospital; d Department of Renal Medicine, Second Affiliated Hospital of Hainan Medical College, Haikou 570311, Hainan Province, China.

**Keywords:** coronavirus disease 2019, meta-analysis, music therapy, protocol

## Abstract

**Background:**

Whether music therapy improves coronavirus disease 2019 (COVID-19) patients’ anxiety, depression, and life quality are still controversial. Therefore, to provide evidence-based medical evidence for clinical non-pharmacological interventions, we performed a meta-analysis of randomized controlled trials of music therapy for COVID-19 patients’ anxiety, depression, and life quality.

**Methods:**

Cochrane Central Register of Controlled Trials Repositories, PubMed, Embase, Web of Science and Chinese Science Citation Database, China National Knowledge Infrastructure, Chinese Biomedical Literature Database, Chinese Scientific Journal Database, and Wan-Fang database were searched to identify studies on the evaluation of the effectiveness of the music-based intervention on COVID-19 patients’ anxiety, depression, and life quality from inception to May 2021. Two researchers independently carried out data extraction and literature quality evaluation of the quality and the meta-analysis on the included literature was performed with Revman5.3 software.

**Results:**

The results of this meta-analysis will be submitted to a peer-reviewed journal for publication.

**Conclusion:**

This study will provide reliable evidence-based evidence for the effects of music therapy on COVID-19 patients’ anxiety, depression, and life quality.

## 1 Introduction

In December 2019, a severe acute respiratory syndrome coronavirus 2, characterized by pneumonia, broke out in Wuhan, China, known as coronavirus disease 2019 (COVID-19)^[1–3]^ that is transmitted from person to person through the respiratory tract and contact with infected people, and all people are generally susceptible.^[4,5]^ The incubation period is generally from 1 to 14 days, and up to now, there exists no effective confirmed antiviral therapy.^[6]^

Prevention and isolation are the 2 most effective protective measures.^[7,8]^ To prevent the wider spread of the epidemic, confirmed cases have been isolated for treatment and suspected cases have been self-quarantined for observation. Isolated people often have psychological stress reactions to various factors.^[9–11]^ When focusing on the prevention and control of COVID-19 clinically, our attention should also be paid to psychological intervention for different groups of people.^[12,13]^

As an interdisciplinary subject, music therapy integrates medicine, music, and psychology together.^[14,15]^ Meanwhile, being a non-drug intervention method, it has been increasingly applied in clinical practice by researchers.^[16,17]^ Therefore, music therapy may be used as a non-pharmacological intervention for psychological intervention in COVID-19 patients. However, there is still insufficient clinical evidence to support it. A meta-analysis was conducted to further evaluate the effects of music therapy intervention on COVID-19 patients’ anxiety, depression, and life quality.

## 2 Methods

### 2.1 Protocol

Under the guidance of the preferred reporting items for systematic reviews and meta-analysis protocols, this protocol of systematic review and meta-analysis has been drafted.^[18]^ The research framework has been registered on the open science framework (OSF) (Registration Number: DOI 10.17605/OSF.IO/VKF8X).

### 2.2 Ethics

Since this is a protocol with no patient recruitment and personal information collection, the approval of the ethics committee is not required.

### 2.3 Eligibility criteria

#### 2.3.1 Types of studies

We will collect all randomized controlled trials.

#### 2.3.2 Participants

COVID-19 patients.

#### 2.3.3 Interventions

Patients in the control group were given routine treatment, while patients in the experimental group accepted music therapy on the basis of routine treatment.

#### 2.3.4 Outcome index

Any rating scale that describes anxiety, depression, and life quality.

### 2.4 Exclusion criteria

(1) Studies without a control group. (2) Review articles, techniques, case reports, letters to the editor, and editorials are excluded.

### 2.5 Search strategy

Computer was used to retrieve Cochrane Central Register of Controlled Trials Repositories, PubMed, Embase, Web of Science and Chinese Science Citation Database, China National Knowledge Infrastructure, Chinese Biomedical Literature Database, Chinese Scientific Journal Database, and Wan-Fang database. The retrieval time limit was between the establishment of the database and May 2021. Taking PubMed as an example, the retrieval strategy is displayed in Table 1.

**Table 1:** PubMed search strategy.

Number	Search terms
#1	Music Therapy [MeSH]
#2	Therapy, Music [Title/Abstract]
#3	or/1–2
#4	Corona Virus [Title/Abstract]
#5	Corona Virus Disease 2019 [Title/Abstract]
#6	COVID-19 [Title/Abstract]
#7	Novel coronavirus [Title/Abstract]
#8	Novel coronavirus pneumonia [Title/Abstract]
#9	or/4–8
#10	#3 AND #9

### 2.6 Data screening and extraction

**Figure 1:**
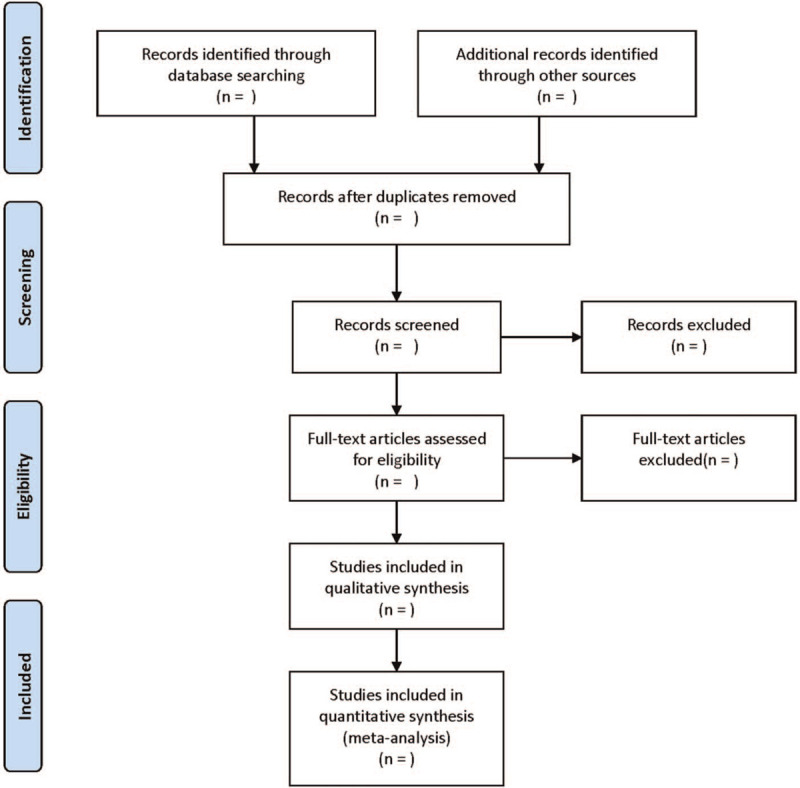
Flow diagram of the literature retrieval.

The literature selection process is listed in Figure 1. Two authors independently searched and screened relevant papers. EndNote X7 software was utilized to delete the duplicates. The titles and abstracts of all searched papers were checked for eligibility. Relevant papers were selected, and then the full-text papers were subsequently assessed by the 2 authors. Finally, a panel meeting was convened for resolving the disagreements about the inclusion of the papers.

We developed a data abstraction form to extract all useful data: (i) the characteristics of papers (authors, publish year, and country); (ii) the characteristics of participators (sample size, mean age, sex ratio, and study period); (iii) study design (random allocation, allocation concealment, masking, selection process of participators, and loss to follow-up); (iv) music therapy process (music therapy method, music therapy period, music therapy frequency, minutes per session, and the treatment measures in the control group); and (v) outcome measures (anxiety, depression, and life quality score).

### 2.7 Quality evaluation

Two authors independently assessed the risk of bias of included studies using Cochrane Collaboration's risk of the bias assessment tool, and all disagreements were resolved by discussing with a third author.

### 2.8 Statistical analysis

Rev-Man 5.3 software was applied for the meta-analysis. The pooled effects were estimated using the standardized mean differences and its 95% confidence interval (95% CI). Heterogeneity between studies was assessed by *I*-square (*I*^2^) and Q-statistic (*P* < .10), and a high *I*^2^ (>50%) was recognized as heterogeneity. If *P* ≥ .1 and *I*^2^ ≤ 50%, there was no statistical heterogeneity among the results of the studies, and a fixed-effect model (Mantel–Haenszel method) was adopted for analysis, otherwise a random-effect model was used.

#### 2.8.1 Dealing with missing data

If there are insufficient or missing data in the literature, the authors will be contacted via email. If the data are still not available, only the currently available data will be analyzed and the potential impacts will be discussed.

#### 2.8.2 Subgroup analysis

According to the duration of intervention and severity of illness, subgroup analysis will be carried out.

#### 2.8.3 Sensitivity analysis

We also performed sensitivity analyses to test the robustness of the results by re-estimating the pooled effects with a fixed-effect or random-effect model.

#### 2.8.4 Publication bias

If the number of studies included in an outcome indicator is no less than 10, a funnel chart will be used to assess publication bias.^[19]^

## 3 Discussion

Clinical observation revealed that COVID-19 patients had different degrees of diarrhea, nausea, decreased appetite, rash, and other adverse reactions during antiviral treatment.^[20,21]^ At the same time, to cutoff the transmission route, the confirmed patients accepted isolation treatment and other prevention and control measures, which leads to anxiety, stress, loneliness, depression, and despair.^[20]^ Excessively negative emotions can result in obsessive thinking, and, in severe cases, psychopathy seriously affects the treatment and recovery of COVID-19 patients. In this study, the principles and methods of evidence-based medicine were applied to evaluate the effectiveness of music therapy, so as to further clarify the effects of music therapy on COVID-19 patients’ anxiety, depression, and life quality to provide a basis for clinical application.

## Author contributions

**Conceptualization:** Jiaqi Huang, Xiaomei Chen, and Haiying Li.

**Data curation:** Jiaqi Huang, Xiaomei Chen, Haiying Li, and Xiaoying Zheng.

**Funding acquisition:** Jiaqi Huang.

**Funding support:** Jiaqi Huang.

**Project administration:** Jiaqi Huang.

**Resources:** Xiaoying Zheng.

**Software operating:** Xiaomei Chen.

**Supervision:** Jiaqi Huang and Xiaoying Zheng.

**Validation:** Xiaoying Zheng.

**Visualization:** Xiaoying Zheng.

**Writing – original draft:** Jiaqi Huang, Xiaomei Chen, and Haiying Li.

**Writing – review & editing:** Jiaqi Huang, Xiaomei Chen, and Haiying Li.
